# Erythema Multiforme-Like Presentation in an Asymptomatic COVID-19 Patient

**DOI:** 10.7759/cureus.20814

**Published:** 2021-12-29

**Authors:** Danial Tahir, Moutasim Souliman, Adrian Mola De La Rosa, Ola Al-Jobory, Tarek Naguib

**Affiliations:** 1 Internal Medicine, Ayub Medical College, Abbottabad, PAK; 2 Internal Medicine, Texas Tech University Health Sciences Center, Amarillo, USA

**Keywords:** suboxone, lyrica, seroquel, citalopram, celexa, oxcarbazepine, mirtazapine, covid19, erythema multiforme

## Abstract

In clinical practice, there are a lot of variations in disease manifestations. Diseases are constantly evolving, and one negative test cannot completely rule out a disease. Erythema multiforme (EM) is a common mucocutaneous disease that can be linked to a lot of etiologies, with the most common being herpes simplex virus (HSV) types 1 and 2, *Mycoplasma pneumoniae*, and the use of various drugs. Severe acute respiratory syndrome coronavirus 2 (SARS-CoV-2) is a new virus, and traditionally it is not the first differential for EM eruptions. We report the case of a 52-year-old female patient with a history of multiple drug use, pneumonia-like symptoms, an initial negative viral panel for SARS-CoV-2, followed by a positive polymerase chain reaction (PCR) test, asymptomatic coronavirus disease 2019 (COVID-19) clinical course, and break-out of typical targetoid lesions of EM. Throughout her hospital stay, the patient maintained her oxygenation levels and improved clinically with steroids and symptomatic treatment. She regained her health and was counseled to quit smoking, alcohol, and opioid usage at the time of discharge from the hospital, and a regular follow-up with her primary care practitioner (PCP) was advised.

## Introduction

Several different causes are attributed to the development of erythema multiforme (EM), including the use of various medications, immunosuppressive states such as malignancies, and steroid use; EM can also affect post-transplant and AIDS patients. However, more than 90% of cases of EM are caused by infectious agents like herpes simplex virus (HSV) types 1 and 2 and *Mycoplasma pneumoniae*. The remaining 10% of cases can be linked to an adverse drug reaction, mostly antibiotics such as sulfonamides, nonsteroidal anti-inflammatory drugs (NSAIDs), and anti-epileptics [1]. Therefore, in patients with EM-like presentation, all probable causes should always be explored. We present a case of a 52-year-old female with multiple medication history, smoking, alcoholism, opioid dependence, and an initial negative coronavirus disease 2019 (COVID-19) polymerase chain reaction (PCR) test. It has been reported that up to 54% of COVID-19 patients can have a subsequent positive COVID-19 PCR test [2]. Most of the COVID-19 infections in the younger population are asymptomatic. A study in Shenzhen, China reported that there were no symptoms in 30.9% of middle-aged (median age: 49 years) patients who were infected with severe acute respiratory syndrome coronavirus 2 (SARS-CoV-2) [3]. Hence, with a high degree of suspicion, the PCR test for SARS-CoV-2 was repeated in our patient, and it came back positive.

## Case presentation

A 52-year-old female, from the northwestern region of Texas, with a history of depression, manic disorder, anxiety with anxiety attacks, tobacco dependence, opiate dependence on Suboxone therapy, alcoholism, and obesity presented to our ER with complaints of raised itchy rash with targetoid lesions, and with clear vesicles covering lower extremity, buttocks, and arms bilaterally. She had been prescribed doxycycline for treating pneumonia-like symptoms; this had been followed by the eruption of the rash, which had started from the lower extremities and moved upwards. The rash had progressively worsened with raised clear vesicles. She had been evaluated by her primary care practitioner (PCP) and advised to stop taking her medications, which included mirtazapine, oxcarbazepine, Celexa (citalopram), Seroquel (quetiapine), Lyrica (pregabalin), Reglan (metoclopramide), Suboxone (buprenorphine) as well as the recently prescribed doxycycline. She had been taking medicines for her psychiatric issues for years, and the rash had started before she began to take doxycycline for her chest complaints. Besides the rash, she also mentioned atypical chest pain that worsened with taking deep breaths and 5/10 non-radiating pressure-like pain that lasted from eight to 10 hours. During her stay in the ER, a chest X-ray showed scarring in the right upper lung; EKG revealed findings of sinus bradycardia, heart rate of 53 beats per minute, with no acute ST or T wave changes. Her urine toxicology screen test was negative in the ER. For her itchy rash, she was given 125 mg IV Solu-Medrol and 50 mg IV hydroxyzine. Her other labs are presented in Table 1.

**Table 1:** Investigation results of the patient ALT: alanine aminotransferase; AST: aspartate aminotransferase; BUN: blood urea nitrogen; GFR: glomerular filtration rate; TSH: thyroid-stimulating hormone

Labs	Result	Normal range
White blood cell count	10,600/μL	4,000–11,000/μL
Hemoglobin	11.3 g/dL	12.0–15.5 g/dL
Platelet count	526,000/μL of blood	150,000–400,000/μL of blood
BUN	11 mg/dL	6–24 mg/dL
Serum creatinine	0.5 mg/dL	0.6–1.1 mg/dL in females
estimated GFR	110.7 ml/min	>90 ml/min
ALT	20 U/L	7–55 U/L
AST	24 U/L	10–40 U/L
TSH	1.63 mIU/L	0.5–5.0 mIU/L
Free T4	0.72 ng/dL	0.9–2.3 ng/dL

**Figure 1:**
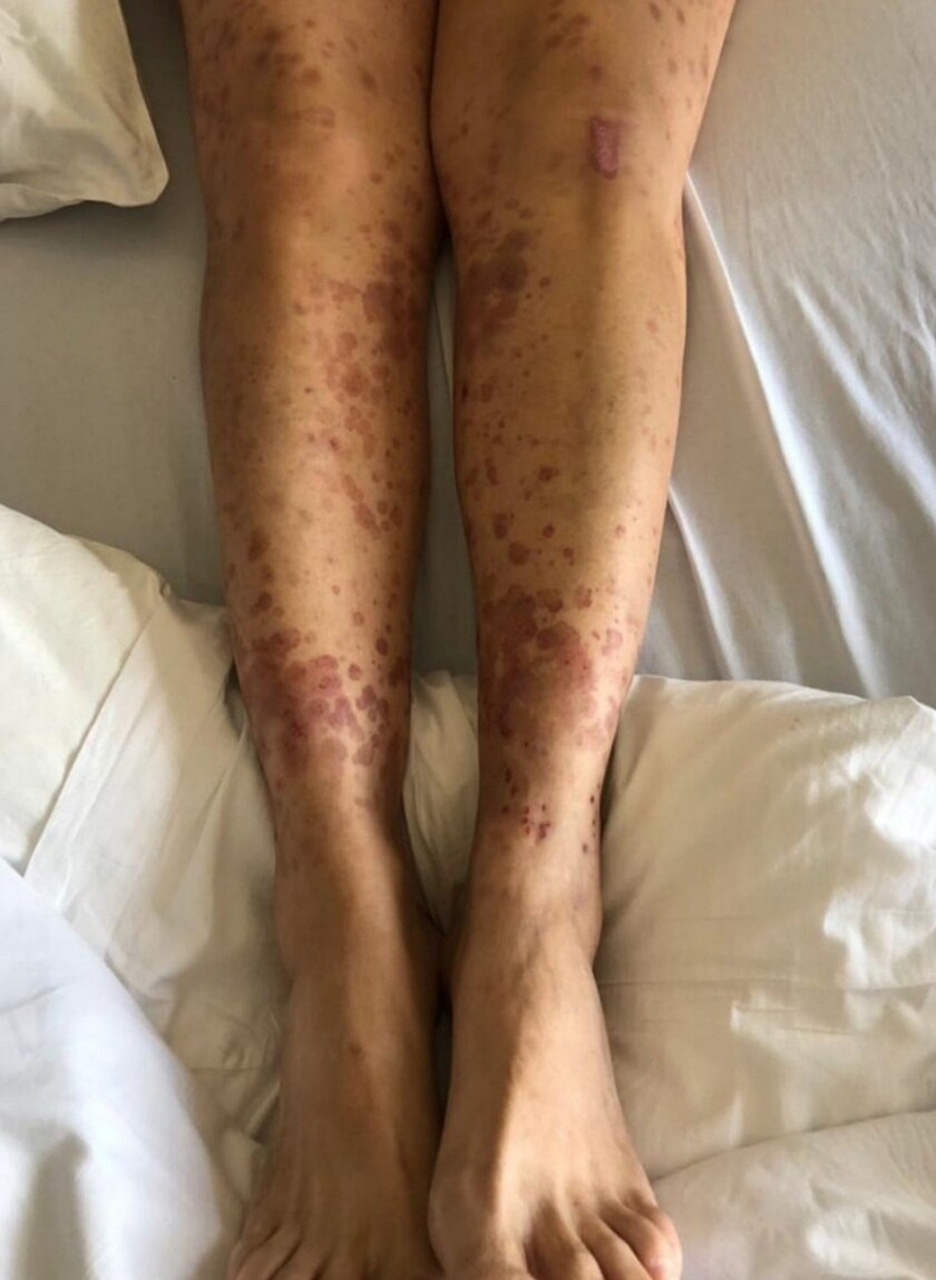
Skin examination of the patient The image shows erythema multiforme-like targetoid lesions on the lower extremities, showing round sharp margins, with three color zones: dark dusky red center, slightly pale pink ring, and outermost bright red-colored ring

Upon admission to the hospital service, she denied any fever, chills, shortness of breath, nausea, vomiting, and any neurological or psychiatric disturbances. Her skin examination showed multiple bilateral target/macular lesions varying in size. The target lesions appeared bigger in size in the lower extremities, as can be seen in the picture below (Figure 1), and extended up to the umbilicus, whereas on the upper extremities, it stretched from the elbow to the wrists and were tiny macular rashes. The figure below shows EM-like skin lesions, with sharp edges, typical spherical contours, and three color zones: dusky or dark red middle crusty blister, followed by the slightly pale pink ring, and an outermost bright red ring.

Initially, her COVID-19 test had been negative, but on repeating it along with the viral panel, it came back positive. She was also tested for *Mycoplasma pneumoniae*, but the IgM and IgG antibodies came back negative. Her C3-C4, antineutrophil cytoplasmic antibodies (ANCA) panel, and antinuclear antibodies (ANA) came back negative, and so did the rapid plasma reagin (RPR) for syphilis, viral serology for viral hepatitides, PCR for HSV DNA, and a non-reactive HIV test. Based on these findings, her EM rash was deemed most likely due to the COVID-19 infection despite the absence of a lot of symptoms.

She was given IV Solu-Medrol 40 mg twice daily. Her rash improved significantly and faded away. She was treated for COVID-19 viral infection associated with systemic inflammatory response syndrome (SIRS) and EM rash and the room air saturation was maintained. She was discharged on a tapered steroid dose, along with Benadryl for itching and famotidine 20 mg twice daily. The patient was counseled to quit smoking and advised a follow-up with her PCP in three to five days.

## Discussion

EM is a skin and mucosal hypersensitivity reaction brought about by various antigenic stimuli and appears as distinctive lesions [4]. The skin and mucosal membrane lesions can be papular, bullous, and necrotic. Commonly, the lesions crop up in 48-72 hours, mostly on the extremities. EM lesions usually heal within one to three weeks [5]. The most frequent etiologies of EM are HSV types 1 and 2 and *Mycoplasma pneumoniae*. Other causes include various bacteria, viruses, fungi, vaccines, and medications like penicillins, cephalosporins, macrolides, and sulfonamides antibiotics [6,7]. EM has been described in patients with all kinds of national backgrounds and ages. However, it is most commonly seen in people between the ages of 20 and 30 years. Men are predisposed to developing EM five times more than women. Patients with immunosuppression, those on steroids, those with systemic lupus erythematosus (SLE), and bone marrow transplant recipients are highly susceptible to EM [8]. Upon analysis, HSV-DNA by PCR test is frequently seen in EM patients. HLA-DQ3 has been widely expressed to be associated with postherpetic EM and can be used as another diagnostic marker. Pathophysiologically, cell-mediated immunity is responsible for the destruction of epithelial cells. Macrophages and CD8 T lymphocytes release a large number of cytokines that lead to the inflammation and subsequent death of epithelial cells [9].

The gold standard for the diagnosis of EM is a punch biopsy. The lesion biopsy will show dermatitis, with the tremendous foray of macrophages and CD8 T lymphocytes at the dermo-epidermal junction, along with dyskeratosis of basal keratinocytes, epidermal necrosis, and subepidermal blisters. Before the eruption of EM skin lesions, there will be fever, malaise, arthralgia, and joint swellings observed in the patients. Typical EM skin lesions have a clear center, enclosed with a blister and red periphery. These lesions do not cause pruritus but an uneasy burning sensation. For mucosal lesions, the most common site is in the mouth. They are also frequently reported in the genital and ocular mucosal membranes. Unlike cutaneous lesions, they can quickly evolve into agonizing erosions. Extensive mucocutaneous lesions can result in dehydration and weight loss. Extracutaneous manifestations include respiratory symptoms with complaints like cough and dyspnea.

The global pandemic caused by SARS-CoV-2 has also been responsible for various dermatological lesions including EM-like eruptions. Different types of medicines have been used for the treatment of COVID-19, and there may be a correlation between EM lesions and the drugs used in the management of deadly viral infections. Therefore, EM-like eruptions that are associated with COVID-19 have been classified into three categories: virus-related juvenile type (patients aged less than 30 years), virus-related older type (patients older than 55 years), and the drug-induced type. So far, the skin lesions have not affected the severity and length of hospital stay due to COVID-19 [10].

A double-blinded randomized clinical trial showed that remdesivir could lead to significant improvement in patients with severe COVID-19 infection. The Kaplan-Meier estimates of mortality were 6.7% with remdesivir and 11.9% with the placebo by the 15th day of hospitalization [11]. However, the eruption of EM-like skin lesions does not necessarily signify a severe COVID-19 infection, and other signs of severity, clinical picture, and diagnostic markers should be assessed before initiating remdesivir in hospitalized patients.

## Conclusions

The development of EM can be attributed to several causes, such as HSV types 1 and 2, *Mycoplasma pneumoniae*, viral hepatitides, malignancies, and usage of various drugs. However, a high degree of suspicion should always be maintained about the evolving etiologies, e.g., SARS-CoV-2. Our patient presented with an initial negative test; however, given the high rate of prevalence of COVID-19 in the community, it should be considered as one of the top differentials. Further studies are required to understand the exact mechanism of how SARS-CoV-2 affects the skin and mucosal cells, as well as how the prognosis and management can be best tailored for viral infections with such cutaneous lesions.

## References

[1] Paulino L, Hamblin DJ, Osondu N, Amini R (2018). Variants of erythema multiforme: a case report and literature review. Cureus.

[2] Pang J, Wang MX, Ang IY (2020). Potential rapid diagnostics, vaccine and therapeutics for 2019 novel coronavirus (2019-nCoV): a systematic review. J Clin Med.

[3] Gao Z, Xu Y, Sun C, Wang X, Guo Y, Qiu S, Ma K (2021). A systematic review of asymptomatic infections with COVID-19. J Microbiol Immunol Infect.

[4] Hafsi W, Badri T (2021). Erythema Multiforme. https://www.ncbi.nlm.nih.gov/books/NBK470259/.

[5] Magri F, Chello C, Pranteda G, Pranteda G (2019). Erythema multiforme: differences between HSV-1 and HSV-2 and management of the disease-a case report and mini review. Dermatol Ther.

[6] Hashemi DA, Carlos C, Rosenbach M (2019). Herpes-associated erythema multiforme. JAMA Dermatol.

[7] Wang Y, Liu P (2017). A case of erythema multiforme drug eruption associated with erythrodermic psoriasis induced by sofosbuvir and daclatasvir. J Clin Pharm Ther.

[8] Trayes KP, Savage K, Studdiford JS (2018). Annular lesions: diagnosis and treatment. Am Fam Physician.

[9] Britnell SR, Willets AE, Vanderman AJ, Woodard CL, Britt RB (2016). Influence of successful chronic hepatitis C virus treatment with ledipasvir/sofosbuvir on warfarin dosing requirements in four veterans. Pharmacotherapy.

[10] Bennardo L, Nisticò SP, Dastoli S (2021). Erythema multiforme and COVID-19: what do we know?. Medicina (Kaunas).

[11] Beigel JH, Tomashek KM, Dodd LE (2020). Remdesivir for the treatment of Covid-19 - final report. N Engl J Med.

